# Lectures on the Treatment of Diseases of the Liver, Spleen and Intestines

**Published:** 1881-08

**Authors:** E. L. Schurly

**Affiliations:** Professor of the Practice of Medicine in Detroit Medical College, late Professor of Materia Medica and Therapeutics in the same college


### Independent Practitioner.

VOL. II.——*August*, 1881.——No. 8.

ARTICLE I.

### LECTURES ON THE TREATMENT OF DISEASES OF THE LIVER, SPLEEN AND INTESTINES.

BY PROF. E. L. SCHURLY, M. D.

Professor of the Practice of Medicine in Detroit Medical College, late Professor of Materia Medica and Therapeutics in the same college—Reported by Hugo Erichsen.

*Hepatalgia.*—(Neuralgia of the liver.)

Pregnant women, and malarial toxæmic persons are apt to have hepatalgia.

*Treatment.*—If patient is constipated we give a cathartic. Muriate of ammonium is also useful. If this fails, opium or morphine will often prove beneficial. Sometimes a laxative is necessary (senna). This is to be followed by a tonic; ferri sulphas, quinia, or strychnia. Pain does *not* follow the diaphraghmatic line.

*Congestion of the Liver.*—Generally this disease causes a structural change. It is ushered in by a chill. Pain may or may not be present. Jaundice generally appears in the course of the disease. Malarial poisoning is another cause of this disease. It is also caused by hard drinking.

*Treatment.*—If you find the stomach sympathizes give a laxative.

If the stomach is in good order an emetic will do much good. The patient should only take barley water, toast, and tea, for 24 hours. In those cases that suffer from malaria, accompanied by diarrhœa, magnesia sulph. Rochelle salts, infusion of columbo or compound tincture of cinchona will prove useful. Hydrastia is also often beneficial in these cases.

In the case of spirit drinkers, the liver swells in all directions, and organic change takes place.

Order a good hot bath and prescribe :

℞. hydrarg, chlor. mit., gr. x.
Pulv. rhei . . gr. v.

Jalap, in twelve (12) grain doses is useful in these cases. Vegetable cholagogues, as podophyllin, leptandrine, juglandin, taraxacum, with or without hydrastin are very good. Diuretics are beneficial in some cases. We may apply iodide of mercury ointment with lard.

*Jaundice.*—Supposed by some to be due to suppression of bile, over-manufacture of bile or transformation of blood into biliverdin. Mechanical jaundice is due to a tumor or an aneurism.

*Jaundice due to want of bile.*—Open all emunctories. Give cathartics and diuretics. Order a hot steam bath, to be taken at night, to be followed by calomel or blue pill in the morning. After the cathartics have acted, we give the diuretics acetate of potassium combined with dram doses of compound tincture of juniper. Fright frequently causes jaundice. It may be a symptom of other diseases.

If cathartics and diuretics fail, we stimulate the liver directly. Calomel with opium or rhubarb can be given for a short time with success.

If the medication is to be continued, podophilin or one to five grains of leptandrine is useful. 30--40 minims of the fluid extract of the last named remedy alone or with some syrups is also very good. Juglandine and taraxacum is efficient. This can not be kept very long, because it will spoil.

℞ Juglandin, gr. xx.
Vini Xerrici.
Ext. taraxaci, fl. āā ℥ j. M.

Euonymine is equally efficacious. All these drugs must be fresh. Podophyllin ought to be given with a carminative, as ginger, whiskey or capsicum.

*Jaundice from Obstruction of the Gall Duct.*—Give only albuminous food; 10--15 grains of inspissated ox gall given one or two hours after meals is useful. Constipation is best relieved by rhubarb, Elimination should be kept up by diaphoretics and diuretics.

*Jaundice due to Gall Stones.*—The passage of gall stone causes terrible pain; this pain is relieved by the hypodermic injection of morphia. If this fails, the internal administration of morphia and dioscorine will prove successful. The dose of dioscorine is 1--4 grs. This may be combined with camphor and opium. Chloroform given cautiously is useful, Give hot baths till syncope sets in. Apply hot fomentations to the epigastrium. Administer ether or chloroform, till the gall stone has passed. The patient is to be nourished by enemata, to which salt is added to aid assimilation. Alkalies are useful; 10-20 grains of phosphate of soda in water is efficacious. Sweet oil alone or in emulsion with egg is very good. Saline mineral waters are indicated. Iron is to be given subsequently for one or two months.

*Inflammation of the Liver* often results in localized or circumscribed abscess. Ten or fifteen leeches may be applied locally. Mercury may be used in syphylitic inflammation. It may also be used as a cathartic, with care. Ipecac in doses of ten or fifteen or even thirty grs., or even ʒ j is very good. Tolerance may be established by commencing with ten or fifteen grains. This treatment is to be continued three days. Tartar emetic is useful in combination with pot. nit., 1-12 of a grain of tartar emetic with 1–2 grs. of potassii nit. Blisters are useful. Hot fomentations are very good. Large poultices or fomentations of linseed oil, slippery elm or bread and water may be employed. Aconite is the best substitute for tartar emetic. Aconite is administered: Tr. aconit. rad., 5 gtt. 3 times a day, just about the end of first stage. Nitro muriatic acid is good after the use of the aconite, blisters, etc. The strong acid can be used, 3–5 min. in a tumblerful of water. Two tumblersful may be drank daily. Inspect the mouth and the pharynx carefully every day. If epithelium comes away, the acid must be stopped. If the inflammation is due to malaria give quinine. In northern climate ten grs., and in southern climate 25 grs. When an abscess has formed stop all treatment. Give nutrients, egg-nogg, etc. The abscess will generally open into the lung, into the intestines or the abdominal cavity. If the abscess points externally use the aspirator. In diffuse abscess use the absorbents, i. e. iodide of potash, iodine, etc.

*Cirrhosis of the Liver*, or Atrophy of the liver, is generally a slow disease. It may be accompanied by jaundice or not. We have all the symptoms of chronic gastric catarrh. T.—Change of climate advisable. Prohibit the use of alcohols and spices. Salt may be allowed, and is sometimes even beneficial. Regular bathing is good. Aperients, especially saline, are to be used. In great constipation give purgatives. In the later stages, when dropsy comes on we give elaterium or pulv. jalapae (Jalap 1 part, bitart pot. 2 pts, dose gr. x 1 ʒ) combined with hyoscyamus. Croton oil is to be regarded as the last resort. Elaterium, dose 1-16 to 1-4 gr., elaterin 1-16 in pill. Broom is sometimes useful. Give ext. Scoparii fl. gtt x increasing to ʒ j. Diuretics are used. The better way is to tap the abdomen. This is done in a line with the linea alba, midway between the umbilicus and the pubes. In the male, draw off the urine before tapping. Use a bandage around the abdomen.

*Acute Yellow Atrophy*, the liver has a waxy appearance. This disease can not be cured. Keep the patient quiet by the use of opium.

*Fatty Liver* may be due to malaria or due to the ingestion of too much fatty food. Caused sometimes by malpractice with cod liver oil. Caused by alcohol. T.—Nitrogenous diet. Avoid all starchy food. Rye bread may be given. Frequent bathing good. A course of training, as boating. Farming is also very efficient. Jaundice may exist as a complication or not. Give very little medicine. Alkalies given as cathartics.

*Cancer of the Liver*. Employ palliative treatment. Aid the digestion and relieve pain. Charcoal is sometimes good. Bismuth and magnesia are useful. Carbolic acid in water or chlorine water. In acid fermentations wash out the stomach. As a laxative we may use phosphate of soda or compound liquorice powder or senna and liq-orice. As a carminative use aquaemeuth pip. As an anodyne, opium or morphia.

*Hydatids of the Liver.* This is a species of tænia enclosed by a cyst wall, which comes from sheep or from the dog. They cause cysts in the liver. T.—Preventative and curative. Destroy the cysts by giving ammoniæ mur. sulphur, or by opening the cysts by aspiration.

DISEASES OF THE SPLEEN.

*Acute Splenitis,* generally the result of malaria or exposure to cold, causing congestion. Continuous fever following. T.—Rest. Put the patient to bed. Relieve the portal circulation. Use some analgesic. We use opium or morphia. The constipating effect of the opium may be overcome by salines. Glauber's salts and pil cath co. Hot fomentations and blisters are good. Ext. colocynth and ext. sennae with Syr. Rhei Co. If this gives no rest, act through the kidneys. Give nitrate of potash with squill. We can give depressants for the fever.

*Chronic Splenitis.*—Advise change of climate. Purgatives are good. Dyspepsia and melancholia sometimes are symptoms of chronic spleenitis. Patient complains of yellow stools. Enlargement of the spleen generally present. In ague-cake we get no acute symptoms. T.—Relieve the mesenteric circulation by giving veg-etable cathartics, as resin jalapae, podophyllin, etc. Improved Pil cath co. useful or podophyllin, euonymin combined with leptan-drine. Sometimes it is necessary to give a tonic. Iron with chiretta bark infusion (℥ iss--quart $H_2O$). The best form here of iron is the tartrate or citrate. Remember the constipating effect of iron. We may use:

℞ Ferri cit. gr. xvi.

Liq. ammon. acet. ℥ ij. M.

*Sig.*—Two teaspoonsful every 3 or 4 hours.

Chalk-eating, which is generally due to hysteria, is sometimes good in diseases of the spleen. We may use the mineral acids with chiretta. In the treatment of children, it is better to use cream of tartar. May paint the outside with Tr. iodine and iron and Syr. Rhei co. We sometimes have abscess. Aspirate.—The best place to aspirate for abscess of the liver or spleen is between the ninth or tenth rib.

*Enlargement of the Spleen.*—This is generally caused by chronic splenitis. T.—Advise change of climate. We generally use quinine, which seems to contract the spleen. Purgation just far enough to relieve the veins. Use aloes or colocynth. Iodine is one of the best remedies here. Give the Sol. Iodinii Co. internally and also externally, if patient can stand it. Iodine seems to assist the tonic action of quinine. Tr. Ferri. and Ung. Plumbi iod. externally. Warm baths, if the patient can stand it, 3 times a week. Iodide of potassium is good. Mercury and blisters should not be used. Quinine, arsenic, iron and strichnine.

| | |
|---|---|
| Quinine, . . . | gr., 1-2. |
| Strychnine, . . | gr., 1-32. |
| Iron, . . . | gr., 1-1½ (sulph. ex.) |

Nitro-muriatic acid useful. Simple friction with the hand or a brush is good. Iron water or Chalybeate Springs (Virginia). If the person is a big, vigorous person, a warm bath is useful. If patient be anæmic and weak, a cold douche or a wet pack (towel) is indicated. Animal food should be withheld. In great fever, a small dose of aconite or spirits altheris nit. Hypodermic injections 2x a week good, if no leucocythæmia exist.

*Splenalgia* (common name side-ache) is caused by violent exercise in the normal condition. It is often present in anæmia and hysteria. Splenotomy is not warranted. It is generally a fatal operation.

*Splenic Leucocythœmia* is caused by malaria. It often remains quiet in the body for years. It sometimes comes on in females with menstruation or pregnancy, the patient remaining weak, after ten or fifteen days, unable to get out of bed. T. Make the woman get out of bed and stay out. Give stimulants—cod liver oil and nutrients; aperients. The change in the blood consists in an increase in the number of white corpuscles. T. Preventative and curative. Preventative—Contract spleen, if large. 2. Better the condition of the blood. 3. Keep up the vitality. 4. Prevent the disease by removing the patient from malarious regions. Keep patient in a condition of supra health. Curative T.—Give vegetable food ; if anæmia is present, give animal food. Quinine should not be persisted in when the stomach is irritable. If it is to be continued we can combine it with iron or strychnine. When we have constipation we give tr. ferri chlor. Ergotine should not be used if the arterioles are contracted by the mouth or hypodermically. Watch for headache or coldness and pain in the extremities. It is best to stop the use of ergotine now and then, and use something else. Ergotine may be used bypodermically twice a week. Piperine and eucalyptus give marked relief. Piperine may be given in doses of—gr. ¼, gr. viii. Use the ext. fl. or tr. eucalyptus. The best form of administration is to take extract eucalyptus fl. with liquorice or syr. of orange. First mix it with spts. rectif.

| | | |
|---|---|---|
| ℞ | Ext. Eucalyptii fl., . . . | ℥ j. |
| | Vini Xerri (vel.), Sptts. Rectif., . . . | ℥ j. |
| | Syr. Glycerine or Orange, gs., . . . | ℥ ij. |
| | *Sig.* teaspoonful 3x a day. | M. |

Mercury should not be used. Faridazation is sometimes very useful. Put the negative pole in front of the body, and the positive pole behind. Cold douche, when applied not too long and with care, is good. Ice is dangerous, and should not be used. Iron should be used as much as possible. Cod liver oil is especially useful with children. Arsenic, given in the form of Fowler's sol. (5 gtt. doses), is very valuable. Give it not longer than five or six days, and then stop. Phosphorus is also very useful. Iodide of iron or potassium can be used with care. Transfusion is useful if it is applied immediately. In hemorrhage, give styptics, as tannic and gallic acid. To check vomiting, use powdered cardamom seed, combined with bismuth. Once in a while ac. hydrobrom can be used, ʒ ss. *well diluted.* This is very useful in nervous patients. In diarrhœa use astringents. Ferri sulph. exic. every two hours. If this fails, inject Monsel's solution per rectum. Oil of turpentine can be given in these cases in ʒ j. doses every four hours, continued for twenty-four hours. In constipation, give mild cathartics. Mild pil. aloes. In dyspnœa, use ammon. carb. or sppts. ammon. Stimulants are given when needed.

*Hutchkin's Disease* consists essentially in an enlargement of splenic cellular tissue. We get progressive œdema. Lymphatic glands become hypertrophied all over the body. Anæmia may be present. The disease is local and general.

T.—Local—Acuapuncture and ice. We generally use iodine or ergot by injection, or iodine and arsenic internally. Phosphorus given with care is very good.

DISEASES OF THE INTESTINES.

*Intestinal Neuralgia*—commonly called colic or bellyache—is generally caused by cold or nervousness. T.—Use opium to relieve pain, either by the mouth or hypodermically. Pil opii et camphoræ.

℞ Pulv. Opii, . . . gr. j.
" Camphoræ, . . gr. ij.
*Sig.*—One every three hours.

Inhalations of chloroform or ether are useful. Hypodermic injections of 1-6 gr. morphiæ, camphor and chlorodyne are useful. Oil of gaultheriæ or peppermint are good.

℞ Tr. Capsicum.
" Opium.
" Camphor.
(Carry in vest pocket with you.)

In persons prone to neuralgia generally, we give iron, phosphorus and arsenic, and other constructive agents. Oil of cajeput (dose gtt. 4-8), given in mucilage gttiij every half hour, is very useful. Dioscorine, in 2–5 gr. doses, in syrup or sherry wine. Apply dry heat to the abdomen. The best way is to heat a plate, cover it by cloth and lay on the abdomen. If bowels are constipated, give pil. cath. co.

*Enteritis*—Generally present in secondary fevers, as typhoid fever, etc.

T.—Very little treatment needed. Give some laxative, as olive or castor oil. Secure rest. Put the patient in a dark, moderately-heated room; allow no conversation to take place; administer no purgative; secure rest by giving opium. After the first stages are over purgation is to be brought on by injections of warm water, etc. We often cannot use opium on account of vomiting. If no vomiting is present, we can even give one grain and one-half. We may give one grain every hour till the desired effect is reached. We use morphia hypodermically, or opium by enema in case vomiting is present. Hot fomentations are good. A cloth rung out in hot water, covered by oiled silk, is best. Ice may be used with care. Citrate of potassium is good. Tamarind infusion is useful. In children, bismuth, with ½ gtt. tr. opii. every two hours. Counter irritants externally. The food is better to be given in enema, with care.

*Constipation* may be caused by obstruction, lack of secretion, atony of muscular layer, too much assimilation, and by habit.

*Due to deficiency of bile.*—T.—The best to use is podophyllin. Simple molasses is good once a day.

*Due to relaxation of rectum (females)*—T.—One-half to one drachm of alum, combined with tr. lobeliæ once a week is excellent. Senna may be used. Exercise after every meal.

*If due to Mesenteric Veins.*—Give aloes, cascara sagrada or stillingia.

| | | |
|---|---|---|
| ℞ | Tr. aloes, ext. stillingiæ fl., . . . | āā ʒ ij. |
| | Tr. nucis vom., . . . . . | ʒ j. M. |
| | *Sig.*—25 drops at night. | |

| | | |
|---|---|---|
| ℞ | Aloes soc., . . . . . | gr. ij. |
| | Ext. colocynth, . . . . . | gr. j. |
| | Ext. nucis vom., ext. belladonæ, | āā gr. ¼. M. |

If hemorrhoids are present we use hamamelis or witchhazel:

| | |
|---|---|
| Witchhazel, . . . | 1-3 parts. |
| Glycerine, . . . | 2-3 " |

In internal hemorrhoids, suppossitories of hamamelis, tannic or gallic acid may be used. Give opium in pain.

| | | |
|---|---|---|
| ℞ | Ext. Hamamel, . . . | gr. v. |
| | Pul. opii, . . . . | gr. x. |
| | Ol. theobromæ, . . . | ℈s. |
| | M. suppossitories, . . | No. x. |

Croton Oil is sometimes of benefit, especially in chronic cases.

| | | |
|---|---|---|
| ℞ | Ol. Tiglii, . . . . . . | gtt. iv. |
| | Ol. Morhuæ, . . . . . . | ℥ ij. |

Injections of cold or tepid water. Common yellow soap whittled into suppository shape introduced into rectum is useful.

*Constipation of Children.*—Soap suppositories. If these are not borne well injection of soap suds may be practiced; corn meal diet. If this fails give: Tr. Aloes and Taraxacum.

*In people* that think they must have a movement of the bowels every day, tell them this is not necessary. Inquire if patent purgatives have been used or not.

*Intussusception.*—(A folding in of the intestinal canal.) Purgatives are generally harmful. Give Enemata of one quart, half a gallon or even one gallon of water or soap suds. If this fails we use it with electricity. Give nux vomica or strychnia by the mouth. Pump or inject air into the intestines. After having the air in, turn patient upside down and turn out the buttocks. This starts the air, which has a kind of an explosive effect on the other end. This should be used last. Belladonna is useful, or atropia and strychnia hypodermically.

*Fæcal Pouch.*—Give citrate of magnesia and follow this up by injections of water and the administration of elaterin.

*Ulceration of the Bowels* is a complication of many diseases. The main indication is to heal the ulcer, to rest the part and prevent rupture. To secure rest, opium and its alkaloids are given. If vomiting is present we give opium hypodermically or by injection. The next best is cannabis indica ; dose ⅙th gr. j., combined with chloral hydrate. We can also combine it profitably with ext. hyoscyami or ext. stramonii. Astringents are useful, especially if diarrhœa is present, tannic or gallic acid, etc. Nitrate of silver is the best for this purpose. Ag. NO. 3 has a special selective action on the mucous membranes and not only on those which are exposed but also on the internal, ¼–½ gr. ext. conii is to be given with it. ⅛–¼–½ gr. of the Ag. NO. 3 may be given. In

*Copious Diarrhœa* we may even give one grain. Give water by the mouth after. Bismuth in large doses ( ʒ ss is the very least) is useful. Carbonate of Bismuth may be combined with phosphate of soda or one of the magnesium salts.

*Diarrhœa* is often the complication of fevers, etc. The so-called summer complaint is endemic.

*Summer Diarrhœa* occurs generally in children. Some cases come on between 3 and 4 in the morning. The disease may be due to sewer-gas or to contamination of drinking water, or bad hygienic surroundings, abuse, etc.

T. Regulate the diet according to hygienic surroundings. The real essence of the trouble is perhaps atmospheric change. Change of climate is advisable, as a trip into the country or on the water, a sea voyage. Do not give a too favorable prognosis. Cold applications are very benefiting. Give a mild alterative. Rheum et creta or lime water. If diarrhœa keeps up give astringents as ext. geranium fl. or Hæmatoxylon. If this fails soda or lime water combined with rhei syr. If copious discharges come on we give 1–2 grs. ac. tannici. In fever give quinine. This can be combined with ext. geranii fl. We may give opium. Tr. deodor or paregoric. Aniseed is useful. If the discharge is white, we use rheum. We may give carminatives if necessary. If the liver is not acting well we may prescribe

| | | |
|---|---|---|
| ℞ | Pulv. opii . . . . . . . | gr. j. |
| | Hyd. chlor. mite . . . . . . | gr. ij. |

Three times a day for one day only.

Advise people that have a dry skin and suffer from diarrhœa to take a Turkish bath.

*Chronic Diarrhœa.*—Ten to fifteen drops of tr. ferri. chlor in water is very useful, three times a day. It may also be given in glycerine. Inf. of Columba combined with tr. geranium or catechu is good. Nux vom. should only be used when the diarrhœa is mucus in character. Pulv. nux vomica is the best for this use. Ipecac is most useful where deficiency of bile is present. Examine the fæces daily. Dandelion is very useful with or without ipecac.

